# Abnormalities in *Clostridioides* and related metabolites before ACTH treatment may be associated with its efficacy in patients with infantile epileptic spasm syndrome

**DOI:** 10.1111/cns.14398

**Published:** 2023-08-08

**Authors:** Lin Wan, Xiuyu Shi, Huimin Yan, Yan Liang, Xinting Liu, Gang Zhu, Jing Zhang, Jing Wang, Mingbang Wang, Guang Yang

**Affiliations:** ^1^ Senior Department of Pediatrics The Seventh Medical Center of PLA General Hospital Beijing China; ^2^ Department of Pediatrics The First Medical Centre, Chinese PLA General Hospital Beijing China; ^3^ Medical School of Chinese People's Liberation Army Beijing China; ^4^ The Second School of Clinical Medicine Southern Medical University Guangzhou China; ^5^ Microbiome Therapy Center, South China Hospital, Medical School, Shenzhen University Shenzhen China; ^6^ Shanghai Key Laboratory of Birth Defects, Division of Neonatology Children's Hospital of Fudan University, National Center for Children's Health Shanghai China; ^7^ Marshall Laboratory of Biomedical Engineering Medical School, Shenzhen University Shenzhen China

**Keywords:** ACTH treatment response, *Clostridioides*, gut microbiota, infantile epileptic spasm syndrome, metabolome

## Abstract

**Objective:**

Adrenocorticotropic hormone (ACTH) is the first‐line treatment of infantile epileptic spasm syndrome (IESS). Its reported effectiveness varies, and our current understanding regarding the role of gut microbiota composition in IESS treatment response is limited. This study assessed the microbiome–metabolome association to understand the role and mechanism of gut microbiota composition in IESS treatment outcomes.

**Methods:**

Children with IESS undergoing ACTH treatment were enrolled. Pre‐treatment stool and serum samples were collected for 16S rRNA gene sequencing and liquid chromatography–tandem mass spectrometry, respectively. The children were divided into “responsive” and “non‐responsive” groups, and gut microbiota and serum metabolome differences were analyzed.

**Results:**

Of the 30 patients with IESS, 14 responded to ACTH and 16 did not. The “non‐responsive” group had larger maleficent *Clostridioides* and *Peptoclostridium*_phage_p630P populations (linear discriminant analysis >2; false discovery rate q < 0.05). Ten metabolites were upregulated (e.g., xanthurenic acid) and 15 were downregulated (e.g., vanillylmandelic acid) (*p* < 0.05). Association analysis of the gut microbiome and serum metabolome revealed that *Clostridioides* and *Peptoclostridium*_phage_p630P2 were positively correlated with linoleic and xanthurenic acids, while *Clostridioides* was negatively correlated with vanillylmandelic acid (*p* < 0.05). A classifier using differential gut bacteria and metabolites achieved an area under the receiver operating characteristic curve of 0.906 to distinguish responders from non‐responders.

**Conclusion:**

This study found significant differences in pre‐treatment gut microbiota and serum metabolome between children with IESS who responded to ACTH and those who did not. Additional exploration may provide valuable information for treatment selection and potential interventions. Our results suggest that varying ACTH responses in patients with IESS may be associated with increased gut *Clostridioides* bacteria and kynurenine pathway alteration, but additional experiments are needed to verify this association.

## Graphical abstract

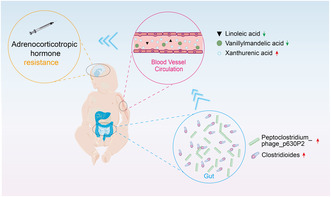

## 1 INTRODUCTION

Infantile epileptic spasm syndrome (IESS) is a distinct category of epilepsy that generally occurs within 2 years of birth and is primarily characterized by spasm clusters. IESS carries a poor prognosis and patients often experience additional neurological effects, such as developmental delay and autism.^1^ Adrenocorticotropic hormone (ACTH) is the first‐line treatment for IESS, and multiple studies report short‐term effectiveness rates ranging from 32% to 64%.^1^, ^2^ Short‐term effectiveness has typically been correlated with the etiology or the timespan between spasm onset and therapy.^1^ However, the specific mechanism underlying ACTH's varying effectiveness has not been elucidated.

The microbiome–gut–brain axis has gradually become recognized as a key factor in the pathogenesis of many diseases, especially neurological disorders, due to the interaction between gut microbiota and the host.^3^, ^4^, ^5^, ^6^, ^7^ Gut microbiota are believed to participate in epilepsy pathogenesis via certain metabolic products^8^, ^9^ by activating the immune system's peripheral inflammatory response. This can occur via a series of mechanisms, including the release of proinflammatory cytokines and chemokines and regulation of neurotransmitters (most notably serotonin, gamma‐aminobutyric acid, and glutamate) that affect the balance between neural network excitation and inhibition.^8^, ^9^, ^10^ In addition, gut microbiota may participate in epilepsy via certain regulatory pathways, including the endocannabinoid system, gut barrier permeability (e.g., by increasing lipopolysaccharide levels), neuroendocrine regulation (e.g., the hypothalamic–pituitary–adrenal axis), and neural pathways (e.g., vagus nerve input and the enteric nervous system).^8^, ^9^, ^10^

Collectively human gut microorganisms possess a gene pool that is 150 times larger than the human genome and includes an extensive collection of enzymes that can metabolize drugs.^11^ Zimmermann et al. assessed the metabolic capacity of 76 diverse human gut bacteria toward 271 oral drugs and revealed that a considerable number of these drugs undergo chemical modification via microbial metabolism.^11^ A study by Javdan et al. revealed that humans each possess a unique gut microbiome that metabolizes drugs at varying rates.^12^ Considerable evidence indicates that differences in gut microbiota and their metabolites may impact drug absorption and metabolism, potentially causing disparities in therapeutic efficacy.^11^, ^12^, ^13^, ^14^ For instance, gut microbiota metabolize levodopa (used to treat Parkinson's disease), thereby potentially decreasing drug bioavailability and increasing adverse effects.^13^ Gut microbiota not only affect drug metabolism, leading to differences in efficacy, but also regulate host metabolism, producing drug resistance. A study by Teng et al. demonstrated the involvement of intestinal microbiome‐mediated nucleotide biosynthesis in response to preoperative neoadjuvant chemoradiotherapy among patients with locally advanced rectal cancer. Specifically, they observed that in patients for whom neoadjuvant chemoradiotherapy was ineffective, genes associated with DNA repair and nucleoside transport were upregulated.^15^

Our previous exploratory study revealed differences in the pre‐treatment gut microbiota composition between patients with “effective” and “ineffective” responses to ACTH treatment.^16^ Based on this observation, we postulated that gut microbiota participate in the regulation of metabolic pathways, thereby affecting the efficacy of ACTH treatment in IESS patients. This study was designed to investigate the link between gut microbiota composition and ACTH treatment efficacy in greater detail. Specifically, this study aimed to (1) determine the differences in gut microbiota composition between infants with IESS who responded well to ACTH therapy and those who did not and (2) explore the potential influence of gut microbiota on ACTH's therapeutic outcomes. Overall, these investigations may enhance our understanding of the complex relationships among gut microbiota composition, metabolic pathways, and treatment efficacy in IESS.

## 2 MATERIALS AND METHODS

### 2.1 Participants

This study enrolled patients with IESS who had undergone ACTH treatment at the Chinese PLA General Hospital between June 2020 and December 2021. The inclusion criteria were as follows: (i) IESS diagnosis based on electroencephalography (EEG) results and clinical characteristics confirmed by two pediatric neurologists according to the criteria of the Commission on Classification and Terminology of the International League Against Epilepsy,^17^ (ii) completion of 14 days of ACTH treatment (2.5 U/kg/d, ≤25 U/d), and (iii) follow‐up of 28 days after discharge. The exclusion criteria were as follows: (i) infection symptom presentation during ACTH infusion (e.g., fever or diarrhea), (ii) antiseizure medication adjustments during ACTH infusion or within 28 days of discharge, (iii) probiotic or antibiotic use in the month preceding enrollment, (iv) fever or diarrhea in the month preceding enrollment, and (v) IESS or other diseases with confirmed metabolic etiology. Clinical data, including sex, age, age at spasm onset, and details regarding antiseizure medicine taken, were collected for all study participants. Informed consent for participation in this study was obtained from the patients' parents.

### 2.2 Fecal and serum samples

Fecal samples were collected on the day of admission and immediately frozen at −80°C for subsequent analysis. On the morning of the day after admission, 3 mL of venous blood was collected before the initiation of ACTH treatment and allowed to stand at 4°C for 2 h. After centrifugation (1500 *g*, 10 min), the supernatant was collected and stored at −80°C until analyzed.

### 2.3 Evaluation of treatment efficacy

Participants were assigned to the “responsive” group if they experienced complete cessation of spasms during ACTH treatment, exhibited no hypsarrhythmia on EEG, and remained spasm free for 28 days after discharge. Participants were assigned to the “non‐responsive” group if they experienced spasms or hypsarrhythmia during ACTH treatment and/or had initial cessation of spasms and hypsarrhythmia but relapsed during the 28 days follow‐up period after discharge.

### 2.4 Sequencing of intestinal microbiota

Fecal samples were used to sequence intestinal microbiota. Overall, the sequencing methods employed were similar to those previously published.^18^ The sequencing sections, paired‐end reads assembly and quality control, and OTU cluster and species annotation can be found in the Supplementary Information file (Supplementary Methods S1).

### 2.5 Preparation for metabolic analysis

Serum samples were used for metabolic analysis, which was performed as previously published.^19^, ^20^, ^21^, ^22^ Detailed descriptions of sample preparation, liquid chromatography conditions, mass spectrum conditions, and data processing are provided in the Supplementary Information file (Supplementary Methods S1).

### 2.6 Analyses

#### 2.6.1 Clinical data statistical analyses

All statistical analyses were performed using SPSS 21.0 (IBM), and statistical significance was set at *p* < 0.05. The data distribution was assessed by the Kolmogorov–Smirnov test. Descriptive data are presented as the mean ± standard deviation or median (with 25th and 75th percentiles). The independent‐samples t‐test was used to identify significant differences in normally distributed data, whereas the Mann–Whitney rank‐sum test was used for non‐normally distributed data. Frequency data were compared using the Chi‐squared test and Fisher's exact probability method. Spearman's test was used to analyze the associations.

#### 2.6.2 Bioinformatics analysis of intestinal microbiota

All analyses were performed using R (version 4.2.2) or QIIME (version 1.9.1) software. The vegan, ggplot, and ape packages in R were employed. Differences in α diversity were calculated using diversity indices (Chao1, Shannon, Simpson, and ACE). In contrast, β‐diversity was determined using weighted UniFrac phylogenetic distance matrices and visualized in principal component analysis (PCA), principal coordinate analysis (PCoA), and non‐metric multidimensional scaling (NMDS) plots. Statistically significant differences in the relative abundances of genera were identified using linear discriminant analysis effect size (LEfSe) and MetaStat™. LDA values >2 or q < 0.05 was considered to be significantly enriched.

#### 2.6.3 Metabolome analysis

The ropls package in R was used for all multivariate data analyses and modeling. Data were mean centered using scaling. Models were constructed based on PCA, orthogonal partial least‐square discriminant analysis (OPLS‐DA), and partial least‐square discriminant analysis (PLS‐DA). Metabolic profiles were visualized using score plots, where each point represents a sample. The corresponding loading plots and S‐plots were generated to provide information on what metabolites were influencing clustering of the samples. All models evaluated were tested for overfitting with methods of permutation tests. The P value, variable importance projection (VIP) produced by OPLS‐DA, and fold change (FC) were applied to discover the contributable variable for classification. Metabolites with *p* values <0.05 and VIP values >1 were considered to be statistically significant.

#### 2.6.4 Gut microbiota–metabolome association analyses

Gut microbiota–metabolome association analyses were performed using Pearson analysis with OmicStudio tools (https://www.omicstudio.cn/tool). *p* < 0.05 and *r* < −0.3 or >0.3 were statistically significant. SPSS 21.0 was used to fit the relevant microbiome and/or metabolome with the logistic regression model. Subsequently, GraphPad software (GraphPad Prism 8.0; GraphPad) was used to draw the receiver operating characteristic (ROC) curve of each classifier. R software and the random forest model were employed to sort variables according to importance in each classifier. Variable importance in each classifier was sorted from high to low according to the value of the mean‐squared error.

## 3 RESULTS

### 3.1 Patient clinical and demographic data

A total of 50 patients with IESS were treated with ACTH at our hospital. Of these, 3 patients had to discontinue ACTH treatment due to infection symptoms, 7 had their anti‐seizure medication (ASM) adjusted within 2 weeks before ACTH treatment, 5 had their ASM adjusted during or after ACTH treatment, and 5 experienced upper respiratory tract infections or diarrhea within a month before commencing ACTH treatment. After excluding these patients, 30 patients with IESS were enrolled (Figure S1).

Among these 30 patients, 12 had unknown etiology, while 18 had a confirmed cause. Among the confirmed cases, 9 had structural causes (7 acquired structural abnormalities and 2 congenital structural abnormalities without identified genetic abnormalities), while 9 were genetically related. Among them, 14 patients exhibited a response to ACTH treatment, while 15 did not respond by the end of ACTH treatment. One patient experienced a relapse within 28 days after completing ACTH treatment without any identifiable triggers and was therefore classified as a non‐responder.

Of the 30 analyzed children, 17 were female and 13 were male. The median age of spasm onset was 4.5 months (25th percentile, 1.75 months; 75th percentile, 10 months), and ACTH treatment was initiated at a median age of 12 months (25th percentile, 8.375 months; 75th percentile, 15 months). The median number of antiepileptic drugs administered was two (25th percentile, 1; 75th percentile, 3). Vigabatrin, valproic acid, and topiramate were the most frequently prescribed medications, with 16, 19, and 25 patients using them, respectively. Twenty‐seven infants were term births, whereas three were pre‐term; nineteen were delivered vaginally, while eleven were delivered by Caesarean section. Among the 30 enrolled participants, no significant differences in sex, age, feeding mode, ASM number or type, etiology (known/unknown), spasm onset age, or delivery type were noted between the two groups (Table 1).

**TABLE 1:** Comparison of demographic and clinical data between the “response” and “no response” groups.

	“Response” group (RBA, *n* = 14)	“No response” group (NRBA, *n* = 16)	*p*‐value
Sex, *n*			0.491^a^
Male	7	6	
Female	7	10	
Delivery pattern, *n*			0.389^a^
Spontaneous	10	9	
Cesarean	4	7	
Feeding pattern, *n*			0.539^b^
Breast	5	5	
Milk	7	6	
Mixed	2	5	
Etiology (known), *n*	7	11	0.296^a^
Number of anti‐seizure medicines	2 (1,3)	2 (1,3)	0.816^c^
Exposure to vigabatrin, *n*	9	7	0.654^a^
Exposure to valproic acid, *n*	7	12	0.156^a^
Exposure to topiramate, *n*	13	12	0.19^b^
Gestational age, *n*			0.626^b^
Mature	13	14	
Premature	1	2	
Developmental delay before spasms onset, *n*	4	9	0.127^a^
Age of spasm onset, months	4 (2, 7.5)	2.5 (6, 11.5)	0.554^c^
Age of ACTH treatment initiation, months	13 (7.25, 15.5)	12 (8.5, 15)	0.744^c^

^a^ Chi‐square test.

^b^ Fisher exact test.

^c^ Rank‐sum test.

### 3.2 Differences in gut microbes between the two groups

Statistical analyses of α‐diversity revealed no significant differences in the Shannon, Simpson, Chao1, or ACE indices between the two groups (Figure S2C–F, *p* < 0.05). PCA, PCoA, NMDS, and the unweighted pair‐group method with arithmetic means all yielded no significant differences between the two groups in β‐diversity (Figure S3, *p* < 0.05).

**FIGURE 1:**
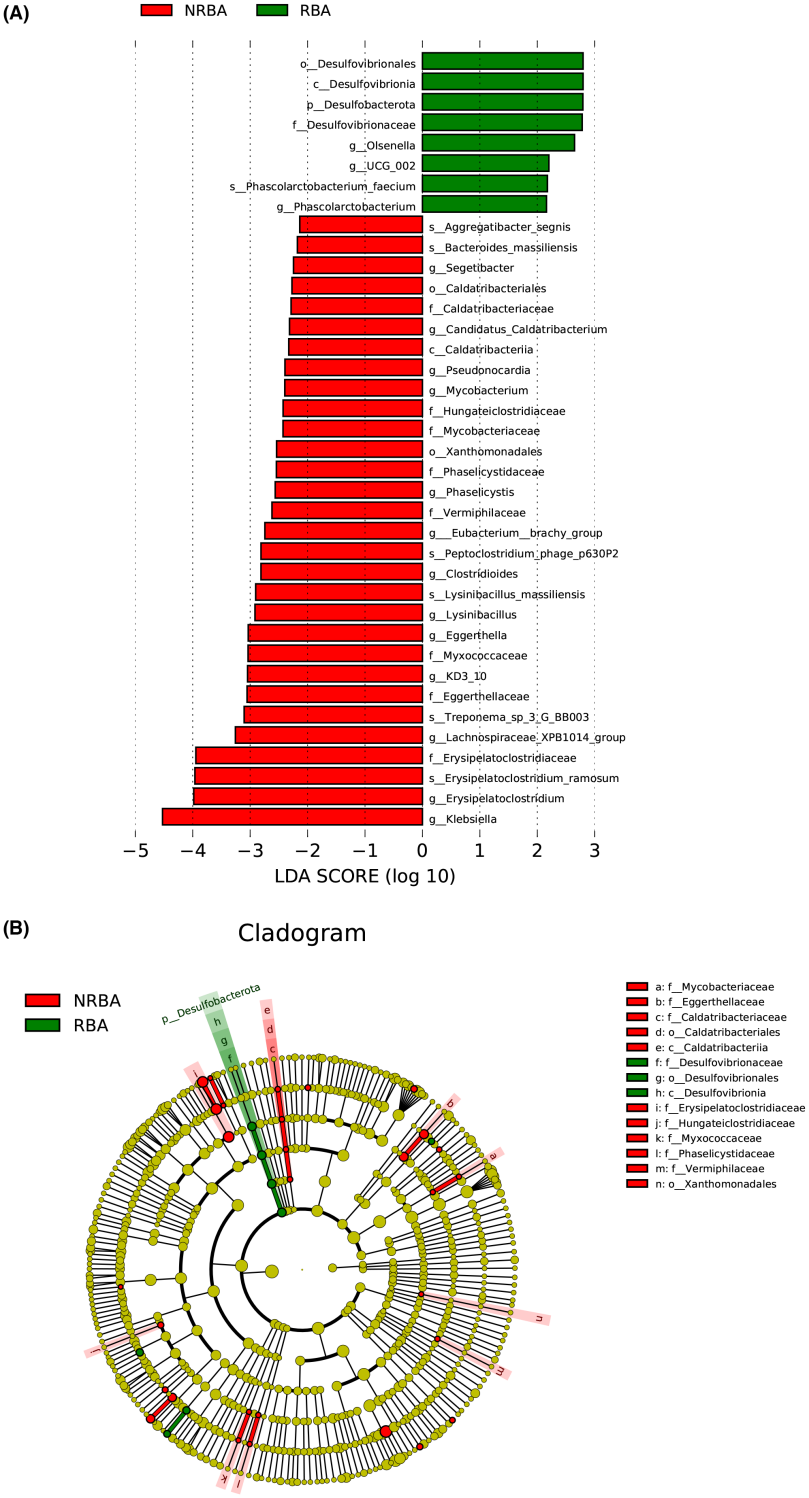
Differences in bacterial genera and species between the “response” and “no response” groups. Red represents the gut microbes that were increased in the ACTH‐non‐responsive group, and green represents the gut microbes that were increased in the ACTH‐responsive group. (analyzed using Lefse; red, “”no response” group, NRBA; blue, “response” group, RBA; p_Phylum, c_Class, g_Genus, o_Order, f_Family, s_Species, LDA >2).

LEfSe analysis revealed significant differences in the relative abundances of genera and species between the two groups. Notably, the genus *Clostridioides* and the species *Peptoclostridium*_phage_p630P2 were more abundant in the non‐responsive group [linear discriminant analysis (LDA) > 2]. In contrast, *Olsenella* and *Phascolarctobacterium* populations were significantly lower in the non‐responsive group (LDA >2) (Figure 1). MetaStat™ analysis also revealed significant differences in the relative abundances of genera and species between the two groups. Again, the genus *Clostridioides* and the species *Peptoclostridium*_phage_p630P2 were more abundant in the non‐responsive group (q < 0.05, Table S1). However, this analysis detected that *Asteroleplasma* and *Lactobacillus ruminis* populations were significantly lower in the non‐responsive group (q < 0.05, Table S1). The intersection of these two analyses found *Clostridioides* and *Peptoclostridium*_phage_p630P2 populations to be significantly increased in the non‐responsive group (q < 0.05 and LDA >2).

### 3.3 Differences in metabolites between the two groups

**FIGURE 2:**
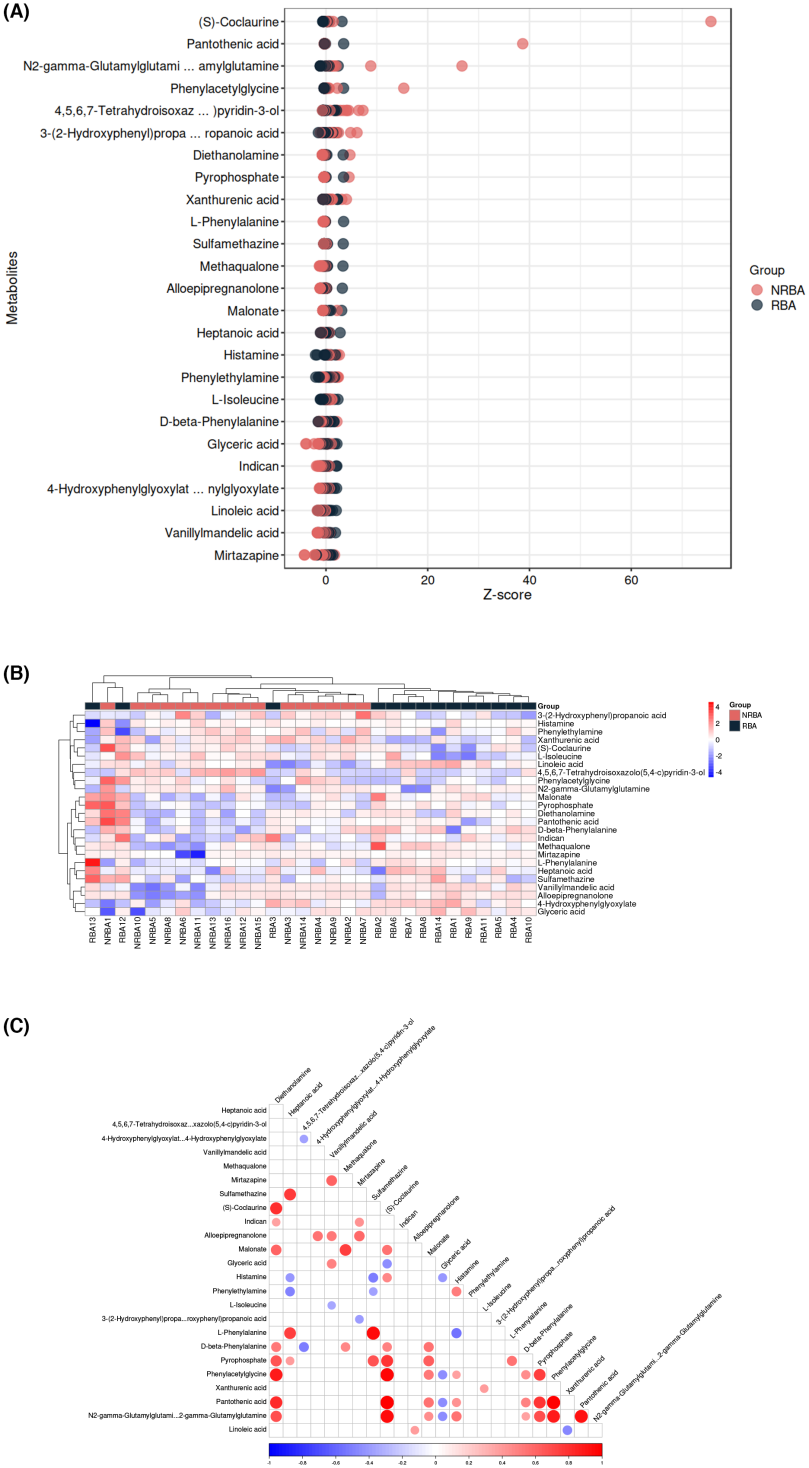
Differences in metabolites between the “response” and “no response” groups. (A) Comparison of specific differential metabolites between the two groups, the abscissa is the value obtained by Z‐score conversion of the relative content of metabolites in the group, and the more to the right represents the more metabolites in the group (red, “no response” group, NRBA; black, “response” group, RBA); (B) Cluster analysis‐based comparison of differential metabolites between the two groups; (red, “no response’” group, NRBA; black, “response” group, RBA); (C) Correlations among metabolites, all the dots represent the significant correlation between the two metabolites; the darker the color, the stronger the correlation; and the larger the dot, the higher the significance; red, positive; blue, negative.

The non‐responsive group exhibited differences in metabolite levels relative to the responsive group, including higher levels of 10 metabolites (L‐isoleucine, histamine, 3‐(2‐hydroxyphenyl) propanoic acid, phenylethylamine, xanthurenic acid, 4,5,6,7‐tetrahydroisoxazolo(5,4‐c)pyridin‐3‐ol, N2‐gamma‐glutamylglutamine, phenylacetylglycine, pantothenic acid, and (S)‐coclaurine) and lower levels of 15 metabolites (sulfamethazine, heptanoic acid, malonate, L‐phenylalanine, 4‐hydroxyphenylglyoxylate, D‐beta‐phenylalanine, linoleic acid, vanillylmandelic acid, allopregnanolone, methaqualone, histamine, mirtazapine, diethanolamine, glyceric acid, and pyrophosphate) (Figure 2A, VIP >1, *p* < 0.05). Cluster analysis revealed that the non‐responsive group (indicated as NRBA in the figure) might exhibit a more consistent metabolic pattern (Figure 2B). Multiple correlations exist among the 25 metabolites (Figure 2C).

### 3.4 Associations between gut microbiota and the serum metabolome

**FIGURE 3:**
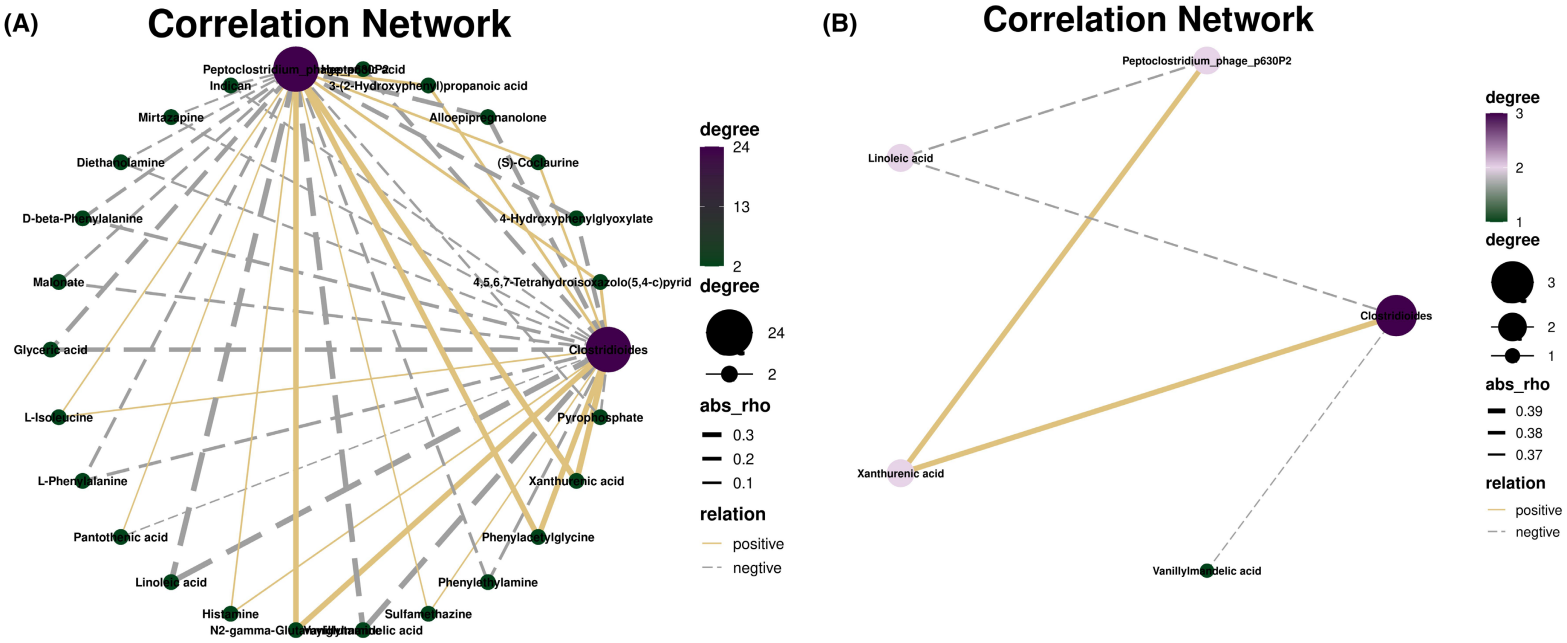
Correlation between the differential microbiota and metabolites in the two groups. (A) Correlation analysis of all different bacteria and metabolites; (B) Statistically significant differences in bacteria and metabolite associations between the two groups (yellow solid line indicates positive correlation, while gray dashed line indicates negative correlation, *r* > 0.3 or < −0.3, *p* < 0.05).

The correlations of *Clostridioides* and *Peptoclostridium*_phage_p630P2 with multiple metabolites were generally consistent (Figure 3A); however, further screening for significance was conducted based on *r* values >0.3 or < −0.3 and *p* < 0.05. The results showed that *Clostridioides* and *Peptoclostridium*_phage_p630P2 were positively correlated with xanthurenic acid, whereas *Clostridioides* and *Peptoclostridium*_phage_p630P2 were negatively correlated with linoleic acid, and *Clostridioides* was negatively correlated with vanillylmandelic (Figure 3B).

### 3.5 Predictability of ACTH response based on pre‐treatment levels of microbiota and metabolites

**FIGURE 4:**
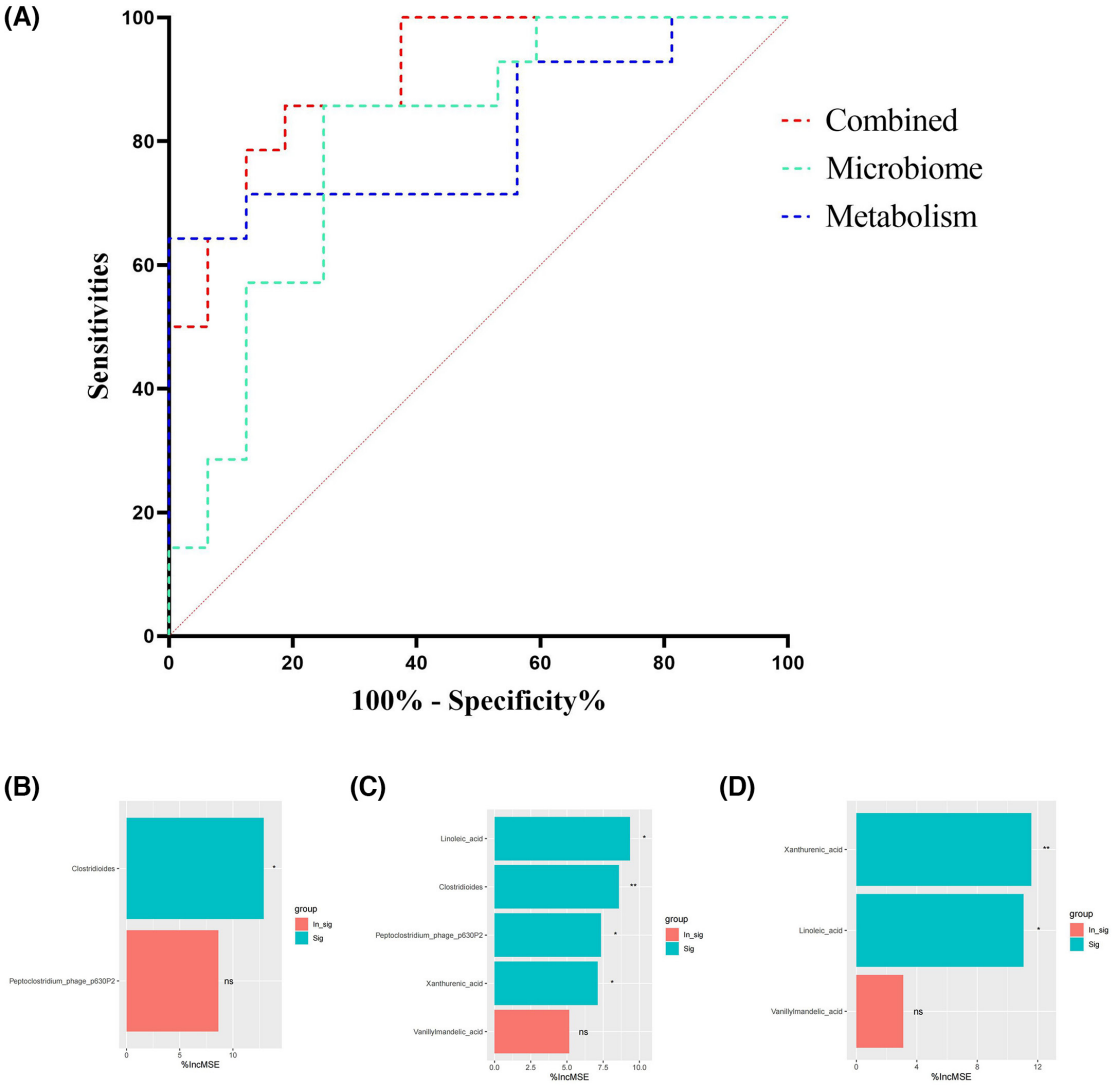
Distinguishing of response to ACTH. (A) Categorization of the 30 patients with IESS based on their response to ACTH treatment using pre‐treatment differential microbiota and/or metabolite data. Microbiome: dotted green, area under the receiver operating characteristic curve (AUC) = 0.8036, 95% confidence interval (CI) = 0.6438, 0.9634; Metabolism: blue dashed line, AUC = 0.8125, 95% CI = 0.6470, 0.9780; Combined: red dotted line, AUC = 0.9063, 95% CI = 0.8037, 1.000; (B) The importance of variants in microbiota classifiers; (C) The importance of variants in metabolism classifiers; D. The importance of variants in gut microbiota–metabolome classifiers (green, significance; red, not significance; *, *p* < 0.05; **, *p* < 0.01; incMSE, increase in mean squared error.

To assess the predictive value of using microbiota levels to distinguish between the two ACTH response groups, the areas under the ROC curves were calculated: 0.8036 [95% confidence interval (CI): 0.6438–0.9634)] and 0.8125 (95% CI: 0.6470–0.9780) when using microbiota‐related differential metabolites (Figure 4A). Nevertheless, discriminative ability improved significantly when the area under the ROC curve for the combination of microbiota and metabolite was calculated: 0.9063 (95% CI: 0.8037–1.000) (Figure 4A). These results demonstrate that the combination of different substances in the two groups effectively distinguished between the two ACTH response groups. Variants were sorted in each classifier according to importance as follows: *Clostridioides* and *Peptoclostridium*_phage_p630P2 in the microbiota classifier; xanthurenic acid, linoleic acid, and vanillylmandelic acid in the metabolite classifier; and linoleic acid, *Clostridioides*, *Peptoclostridium*_phage_p630P2, xanthurenic acid, and vanillylmandelic acid in the gut microbiota–metabolome classifier (Figures 4B–D).

## 4 DISCUSSION

Gut microbiota have been associated with drug resistance in patients with diseases such as breast cancer (chemotherapy drug resistance) and diabetes (insulin resistance), or with lower efficacy of drugs such as levodopa (abnormal metabolism).^13^, ^19^, ^20^ In addition, multiple studies have reported significant differences in the composition of gut microbiota between drug‐resistant and drug‐sensitive patients with epilepsy. For instance, *Bacteroidetes* has been observed predominantly in drug‐sensitive patients,^21^ whereas *Firmicutes* is significantly increased in drug‐resistant patients.^23^, ^24^ While these studies reported differences in the therapeutic efficacies of antiepileptic drugs, they primarily focused on adults. In our earlier study, we identified a significant difference in the relative abundance of gut microbiota between children with IESS who responded to ACTH treatment and those who did not. The present study's findings corroborate our previous results by revealing a significant increase in pre‐treatment levels of *Clostridioides* (a taxon within the *Firmicutes* phylum) in children with IESS who were non‐responsive to ACTH. In addition, we discovered significant pre‐treatment metabolic differences in IESS patients with varying responses to ACTH. The results of two omics correlation analyses suggested that differences in pre‐treatment levels of gut bacteria and metabolites may have predictive value for ACTH treatment efficacy.

Currently, mechanism of action for ACTH treatment in patients with IESS is not well understood. Our previous study suggested that ACTH may alleviate spasms by inhibiting the release of corticotrophin‐releasing hormone (CRH) via negative feedback.^25^ Previous research has indicated that *Clostridium difficile* toxin A can significantly increase the expression of CRH and its receptors CRH1 and CRH2 in the mouse jejunum.^26^ In our study, the *Peptoclostridium*_phage_p630P2 species that we identified belong to the *Clostridioides* genus. Therefore, we speculate that the higher levels of *Clostridioides* observed in the non‐responsive group might have increased CRH production. As a result, the negative feedback effect of peripherally administered ACTH could not effectively alleviate this situation.

The kynurenine pathway is the primary metabolic pathway of tryptophan, and previous studies have confirmed the role of abnormal kynurenine metabolism in the onset of seizures.^27^, ^28^, ^29^, ^30^ Żarnowska et al. found kynurenine metabolism to be significantly altered in treatment‐responsive children with IESS on a ketogenic diet containing lower kynurenine concentrations and higher kynurenic acid concentrations.^28^ Mu et al. demonstrated that a ketogenic diet may affect kynurenine metabolism by regulating gut microbiota. They found that a ketogenic diet or oral antibiotics could alter gut microbiota, regulate kynurenine metabolism, and control spasms in an IESS mouse model.^30^ A study by Yan et al. detected abnormal metabolism of kynurenine pathway‐related metabolites in the cerebrospinal fluid of children with IESS and proposed that the ratio of kynurenic acid to kynurenine could serve as a biomarker for sensitivity to hormone treatment.^31^ These studies collectively suggest that abnormalities in kynurenine metabolism are potentially involved in the development of epilepsy and infantile spasms. Xanthurenic acid is a metabolic product of the kynurenine pathway and is produced from kynurenine by kynurenine 3‐monooxygenase and subsequently converted to xanthurenic acid by kynurenine aminotransferases. In our study, we found a significant increase in xanthurenic acid in the ACTH non‐responsive group, indicating that abnormalities in the kynurenine pathway may be more severe in these patients, consistent with the findings of Yan et al. Additionally, we found a positive correlation between *Clostridioides* and xanthurenic acid levels. Previous studies have shown that *Clostridioides* regulates kynurenine metabolism.^32^ Therefore, we hypothesize that *Clostridioides* affects xanthurenic acid levels by regulating kynurenine metabolism, thereby lowering patient responsiveness to ACTH.

Linoleic acid is a polyunsaturated fatty acid, and previous studies have suggested that it has a protective effect against seizures. Ekici et al. found that linoleic acid combined with conventional antiepileptic drugs had a positive effect on generalized epilepsy.^33^ In a recent meta‐analysis, Asadi‐Pooya et al. found the addition of linoleic acid was beneficial in controlling seizures.^34^ In our study, we found that the non‐responsive group had lower levels of linoleic acid, a phenomenon that might be related to poor spasm control. Stewart Sr. et al. demonstrated that *Clostridioides* can regulate the linoleic acid metabolic pathway.^35^ Moreover, Bruder et al. demonstrated that oxidative metabolites of linoleic acid can stimulate adrenal cells in rats to produce corticosterone and amplify the positive feedback effect of ACTH.^36^ Montero et al. confirmed that linoleic acid can promote the positive feedback effect of ACTH, leading to cortisol release.^37^ Cortisol, in turn, provides negative feedback to inhibit CRH release, and the clinical efficacy of cortisol treatment for IESS has been validated.^38^ Therefore, we speculate that the higher levels of *Clostridioides* in the non‐responsive group might have contributed to the concomitant lower levels of linoleic acid, attenuating the ability of linoleic acid to increase the effect of ACTH on corticosterone, leading to ACTH treatment failure.

**FIGURE 5:**
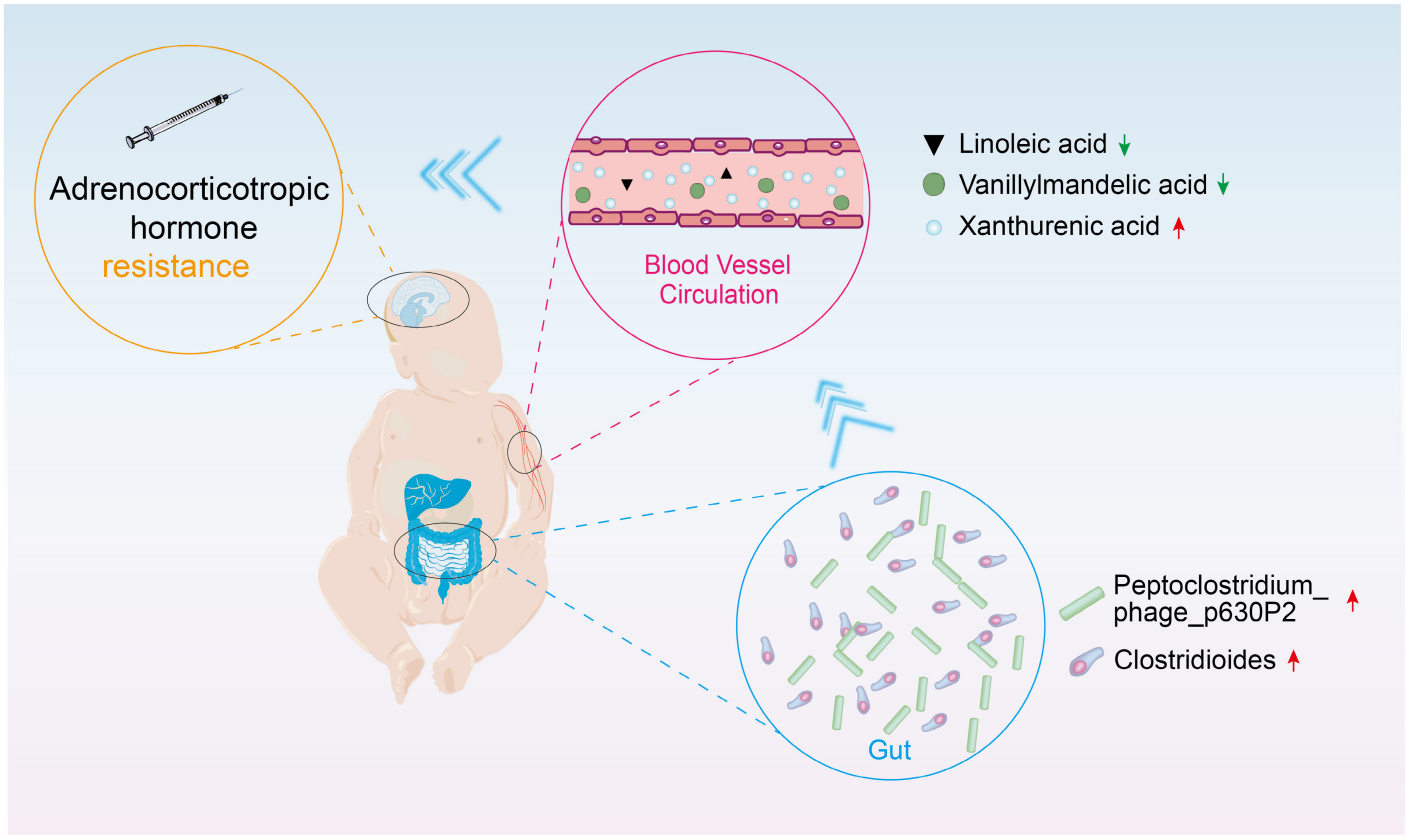
Hypothesized mechanism diagram: Higher levels of *Clostridioides* in the gut of patients with IESS who are non‐responsive to ACTH may contribute to metabolic changes in the host, such as an increase in xanthurenic acid and decreases in linoleic acid and vanillylmandelic acid, ultimately resulting in ACTH treatment failure.

We also observed lower levels of vanillylmandelic acid, the final product of catecholamine metabolism, in the non‐responsive group. This suggests that catecholamine turnover is lower in the non‐responsive group. Catecholamines are an important group of neurotransmitters, and their abnormal metabolism potentially leads to epilepsy.^39^ Reports of seizures following the administration of reuptake catecholamine inhibitors, such as tramadol, are well established in clinical practice.^40^ *Clostridioides* has been reported to significantly affect catecholamine metabolism within the intracranial region of mice and exhibited significantly reduced dopamine beta‐hydroxylase (DBH) activity.^41^ Our study found a negative correlation between *Clostridioides* and vanillylmandelic acid levels (i.e., as the former increased, the latter decreased) in the non‐responsive group. This suggests that elevated levels of *Clostridioides* can disrupt host catecholamine metabolism, leading to a decrease in ACTH treatment efficacy (Figure 5).

Alterations in gut microbiota influence various pathways that contribute to neuronal hyperexcitability and neuroinflammation in epilepsy and similar neurological conditions.^42^ Peng et al. found that gut microbiota composition in drug‐sensitive patients with epilepsy resembled that of healthy individuals; however, a significant difference in microbiota composition was observed in drug‐resistant patients with epilepsy relative to healthy individuals or drug‐sensitive patients with epilepsy.^23^ Similarly, Gong et al. demonstrated that gut microbiota can serve as an effective biomarker for drug response.^43^ A recent review highlighted the potential application of the gut microbiome as a biomarker in the diagnosis and treatment of epilepsy.^44^ Our study was able to effectively differentiate between the patients who were responsive and non‐responsive to ACTH based on differences in gut microbiota and host metabolites. These findings are consistent with previous research and provide valuable insight into future clinical and translational research.

### 4.1 Limitations

This study has several limitations. First, the number of participants enrolled was small. Second, although differences in gut microbiota and blood metabolites were observed, caution is required in drawing conclusions owing to the small study population. Third, it was an observational study, and no specific experiments were conducted to evaluate the observed differences; therefore, additional prospective studies are required to verify the conclusions drawn from our research. Fourth, investigating ACTH treatment‐induced changes in gut microbiota and metabolites may identify better predictive biomarkers ACTH response in children with IESS, and our team is currently engaged in such studies. Finally, whether metabolic abnormalities arise from the patient or changes in gut microbiota composition remains unclear.

## 5 CONCLUSION

In summary, we found that non‐responsive patients have higher levels of *Clostridioides* and different serum metabolites before ACTH treatment than patients who respond to ACTH. The associations between *Clostridioides* and xanthurenic acid, linoleic acid, and vanillylmandelic acid are significant. Therefore, we hypothesize that these differences in microbiota and metabolites are the reason for the difference in treatment efficacy. High levels of *Clostridioides* in the gut of non‐responsive patients may create ACTH resistance by modulating host metabolism. Additionally, these microorganisms and metabolites may serve as biomarkers for a patient's therapeutic response to ACTH therapy and aid in the development of new treatment strategies. However, these conclusions are speculative and require validation by additional studies.

## AUTHOR CONTRIBUTIONS

Lin Wan: Writing – Original Draft, Writing – Review & Editing, Methodology, Software, Formal analysis, Data Curation, and Visualization. Xiuyu Shi: Writing – Original Draft, Resources, Investigation, Validation, Writing ‐ Review & Editing, and Funding acquisition. Huimin Yan: Investigation, Resources, and Data Curation. Yan Liang: Formal analysis, Validation, and Visualization. Xinting Liu: Software and Resources. Gang Zhu: Resources and Visualization. Jing Zhang: Formal Analysis and Resources. Jing Wang: Resources and Methodology. Mingbang Wang: Conceptualization, Writing – Review & Editing, Methodology, Software, Funding Acquisition, and Supervision. Guang Yang: Conceptualization, Methodology, Writing – Review & Editing, Validation, Funding Acquisition, and Project Administration. All authors helped to revise the manuscript with respect to crucial intellectual content. All authors approved the final version for publication.

## FUNDING INFORMATION

This research was funded by the General Project of the Beijing Natural Science Foundation (Reference: 7222187); National Natural Science Foundation of China (Program no.81771389, 82071733); Shenzhen Science, Technology and Innovation Commission (No. JCYJ20220530154601004); Shanghai Talent Development Funding (No. 2020115); BINC Foundation of Nutrition and Care of Maternal & Child (Reference: 2021BINCMCF030); Epilepsy Research Foundation of the Chinese Association Against Epilepsy (Reference: CU‐B‐2021‐11); and the Special Scientific Research Project of Military Family Planning (Reference: 22JSZ20).

## CONFLICT OF INTEREST STATEMENT

No financial or non‐financial benefits were received or will be received from any party directly or indirectly related to the subject of this article. We confirm that we have read the journal's position regarding issues involving ethical publication and affirm that this report is consistent with those guidelines.

## Supporting information


Data S1.

## Data Availability

The original sequencing data used to support this study's findings are restricted by the Ethics Committee of the First Medical Center of the PLA General Hospital to protect patient privacy. Data are available from the corresponding author Guang Yang (yangg301@126.com) for researchers who meet the criteria for access to confidential data.
