# Supplementary material for: Abnormalities in Clostridioides and related metabolites before ACTH treatment may be associated with its efficacy in patients with infantile epileptic spasm syndrome
Source: CNS Neurosci Ther. 2023 Aug 8;30(1):e14398. doi: 10.1111/cns.14398 (PMC10805391; doi:10.1111/cns.14398)
Supplement: Supplementary file 1 — Data S1. [file CNS-30-e14398-s001.zip › CNS new Supplyment.docx]

**Methods**

**Sequencing and analysis of intestinal microbiota**

**1.Sequencing**

**1.1.** **Extraction** **of** **genome** **DNA**

Total genome DNA from fecal samples was extracted using CTAB method. DNA concentration and purity was monitored on 1% agarose gels. According to the concentration, DNA was diluted to 1ng/µL using sterile water.

**1.2.** **Amplicon** **Generation**

16S rRNA genes of distinct regions(16S V4) were amplified used specific primer(515F- 806R) with the barcode. All PCR reactions were carried out with 15 µL of Phusion® High -Fidelity PCR Master Mix (New England Biolabs); 2 µM of forward and reverse primers, and about 10 ng template DNA. Thermal cycling consisted of initial denaturation at 98℃ for 1 min, followed by 30 cycles of denaturation at 98℃ for 10 s, annealing at 50℃ for 30 s, and elongation at 72℃ for 30 s. Finally 72℃ for 5 min.

**1.3.** **PCR** **Products** **quantification** **and** **qualification**

Mix same volume of 1X loading buffer (contained SYB green) with PCR products and operate electrophoresis on 2% agarose gel for detection. PCR products was mixed in equidensity ratios. Then, mixture PCR products was purified with Qiagen Gel Extraction Kit(Qiagen, Germany).

**1.4.** **Library** **preparation** **and** **sequencing**

Sequencing libraries were generated usingTruSeq® DNA PCR-Free Sample Preparation Kit (Illumina, USA) following manufacturer's recommendations and index codes were added. The library quality was assessed on the Qubit@ 2.0 Fluorometer (Thermo Scientific) and Agilent Bioanalyzer 2100 system. At last, the library was sequenced on an Illumina NovaSeq platform and 250 bp paired-end reads.

1. **Paired-end reads assembly and quality control**

**2.1 Data split**

Paired-end reads was assigned to samples based on their unique barcode and truncated by cutting off the barcode and primer sequence.

**2.2 Sequence assembly**

Paired-end reads were merged using FLASH (V1.2.7,http://ccb.jhu.edu/software/FLASH/)[1], a very fast and accurate analysis tool, which was designed to merge paired-end reads when at least some of the reads overlap the read generated from the opposite end of the same DNA fragment, and the splicing sequences were called raw tags.

**2.3 Data Filtration**

Quality filtering on the raw tags were performed under specific filtering conditions to obtain the high-quality clean tags[2] according to the QIIME(V1.9.1, http://qiime.org/scripts/split_libraries_fastq.html)[3] quality controlled process.

**2.4 Chimera removal**

The tags were compared with the reference database(Silva database (16S), https://www.arb-silva.de) using UCHIME Algorithm (http://www.drive5.com/usearch/manual/uchime_algo.html)[4] to detect chimera sequences, and then the chimera sequences were removed [5]. Then the Effective Tags finally obtained.

1. **OTU** **cluster** **and** **Species** **annotation**

**3. 1 OTU Production**

Sequences analysis were performed by Uparse software (Uparse v7.0.1001, http://drive5.com/uparse/)[6]. Sequences with ≥97% similarity were assigned to the same OTUs. Representative sequence for each OTU was screened for further annotation.

**3.2 Species annotation**

For each representative sequence, the Silva Database (http://www.arb-silva.de/)[7] was used based on Mothur algorithm to annotate taxonomic information.

**3.3 Phylogenetic relationship Construction**

In order to study phylogenetic relationship of different OTUs, and the difference of the dominant species in different samples(groups), multiple sequence alignment were conducted using the MUSCLE software (Version 3.8.31，http://www.drive5.com/muscle/)[8].

**3.4 Data Normalization**

OTUs abundance information were normalized using a standard of sequence number corresponding to the sample with the least sequences. Subsequent analysis of alpha diversity and beta diversity were all performed basing on this output normalized data.

**3.5 Species richness analysis**

Rarefaction-curve construction involves randomly extracting a specific amount of sequencing data from the sample, tallying the number of species in operational taxonomic units (OTUs), and plotting a curve based on the extracted sequencing data and corresponding species count. The dilution curve serves as an indicator for assessing the rationality of sequencing data and indirectly reflects species diversity in the sample. As the curve flattens out, it indicates that sufficient sequencing data have been obtained, and additional data will not yield significant results. The rarefaction curve revealed no significant difference in the number of gut-microbiota species between the two groups and sufficient affluence to conduct the subsequent analysis (Fig. S2a). The species accumulation boxplot is an analysis that describes the increase in species diversity with increasing sample size. It was used to evaluate sample-size adequacy and estimate species richness. It produced a relatively smooth curve (Fig. S2b), indicating that the current sample size was sufficient for the subsequent analysis of species abundance.

**Metabolism analysis**

1. **Sample preparation**

LC-MS grade methanol (MeOH) was purchased from Fisher Scientific (Loughborough, UK). 2-Amino-3-(2-chloro-phenyl)-propionic acid was obtained from Aladdin (Shanghai, China). Ultrapure water was generated using a Milli-Q system (Millipore, Bedford, USA).

High-speed centrifuge was obtained from Hunan Xiangyi Experiment Equipment Co., Ltd. (Hunan, China). Centrifugal vacuum evaporator was from Eppendorf China Ltd. (Shanghai, China). Vortex mixer was obtained from Haimen Kylin-bell Lab Instruments Co., Ltd. (Haimen, China). Microporous membrane filters (0.22 µm) was purchased from Tianjin Jinteng Experiment Equipment Co., Ltd. (Tianjin, China).

Thaw the serum sample at 4 ℃, vortex the sample for 1 min after thawing, andmix evenly; Accurately transfer an appropriate amount of sample into a 2 mL centrifuge tube; Add 400 µL methanol (stored at -20 ℃) and vortex for 1 min; Centrifuge for 10 min at 12,000 rpm and 4 °C,take all the supernatant transfer it to a new 2 mL centrifuge tube, concentrate and dry it; Add 150 µL of 2-chloro-l-phenylalanine (4 ppm) solution prepared with 80% methanol water (stored at 4 ℃) to re dissolve the sample ,filter the supernatant by 0.22 μm membrane and transfer into the detection bottle for LC-MS detection[9].

1. **Liquid chromatography conditions**

LC-MS grade acetonitrile (ACN) was purchased from Fisher Scientific (Loughborough, UK). Formic acid was obtained from TCI (Shanghai, China). Ammonium formate was obtained from Sigma-Aldrich (Shanghai, China). Ultrapure water was generated using a Milli-Q system (Millipore, Bedford, USA).

The LC analysis was performed on a ACQUITY UPLC System (Waters, Milford, MA, USA). Chromatography was carried out with an ACQUITY UPLC ® HSS T3 (150×2.1 mm, 1.8 µm) (Waters, Milford, MA, USA). The column maintained at 40 ℃. The flow rate and injection volume were set at 0.25 mL/min and 2 μL, respectively. For LC-ESI (+)-MS analysis, the mobile phases consisted of (C) 0.1% formic acid in acetonitrile (v/v) and (D) 0.1% formic acid in water (v/v). Separation was conducted under the following gradient: 0~1 min, 2% C; 1~9 min, 2%~50% C; 9~12 min, 50%~98% C; 12~13.5 min, 98% C; 13.5~14 min, 98%~2% C; 14~20 min, 2% C. For LC-ESI (-)-MS analysis, the analytes was carried out with (A) acetonitrile and (B) ammonium formate (5mM). Separation was conducted under the following gradient: 0~1 min, 2%A; 1~9 min, 2%~50%A; 9~12 min, 50%~98%A; 12~13.5 min, 98%A; 13.5~14 min, 98%~2%A; 14~17 min, 2% A[10].

1. **Mass spectrum conditions**

Mass spectrometric detection of metabolites was performed on Q Exactive(Thermo Fisher Scientific, USA) with ESI ion source. Simultaneous MS1 and MS/MS (Full MS-ddMS2 mode, data-dependent MS/MS) acquisition was used. The parameters were as follows: sheath gas pressure, 30 arb; aux gas flow, 10 arb; spray voltage, 3.50 kV and -2.50 kV for ESI(+) and ESI(-), respectively; capillary temperature, 325 ℃; MS1 range, m/z 81-1000; MS1 resolving power, 70000 FWHM; number of data dependant scans per cycle, 10; MS/MS resolving power, 17500 FWHM; normalized collision energy, 30%; dynamic exclusion time, automatic[11].

1. **Data processing and multivariate analysis**

The raw data were firstly converted to mzXML format by MSConvert in ProteoWizard software package (v3.0.8789)[12] and processed using XCMS[13] for feature detection, retention time correction and alignment. The metabolites were identified by accuracy mass (< 30 ppm) and MS/MS data which were matched with HMDB[14] (http://www.hmdb.ca), massbank[15] (http://www.massbank.jp/), LipidMaps[16] (http://www.lipidmaps.org), mzcloud[17] (https://www.mzcloud.org) and KEGG[18] (http://www.genome.jp/kegg/). The robust LOESS signal correction (QC-RLSC)[11]was applied for data normalization to correct for any systematic bias. After normalization, only ion peaks with relative standard deviations (RSDs) less than 30 % in QC were kept to ensure proper metabolite identification.

**References**

1. Magoč T, Salzberg SL. FLASH: fast length adjustment of short reads to improve genome assemblies. *Bioinformatics* 2011;**27**:2957-2963.

2. Bokulich NA, Subramanian S, Faith JJ, et al. Quality-filtering vastly improves diversity estimates from Illumina amplicon sequencing. *Nat Methods* 2013;**10**:57-59.

3. Caporaso JG, Kuczynski J, Stombaugh J, et al. QIIME allows analysis of high-throughput community sequencing data. *Nat Methods* 2010;**7**:335-336.

4. Edgar RC, Haas BJ, Clemente JC, Quince C, Knight R. UCHIME improves sensitivity and speed of chimera detection. *Bioinformatics* 2011;**27**:2194-2200.

5. Haas BJ, Gevers D, Earl AM, et al. Chimeric 16S rRNA sequence formation and detection in Sanger and 454-pyrosequenced PCR amplicons. *Genome Res* 2011;**21**:494-504.

6. Edgar RC. UPARSE: highly accurate OTU sequences from microbial amplicon reads. *Nat Methods* 2013;**10**:996-998.

7. Quast C, Pruesse E, Yilmaz P, et al. The SILVA ribosomal RNA gene database project: improved data processing and web-based tools. *Nucleic Acids Res* 2013;**41**:D590-596.

8. Edgar RC. MUSCLE: multiple sequence alignment with high accuracy and high throughput. *Nucleic Acids Res* 2004;**32**:1792-1797.

9. Demurtas A, Pescina S, Nicoli S, Santi P, Ribeiro de Araujo D, Padula C. Validation of a HPLC-UV method for the quantification of budesonide in skin layers. *J Chromatogr B Analyt Technol Biomed Life Sci* 2021;**1164**:122512.

10. Zelena E, Dunn WB, Broadhurst D, et al. Development of a robust and repeatable UPLC-MS method for the long-term metabolomic study of human serum. *Anal Chem* 2009;**81**:1357-1364.

11. Want EJ, Masson P, Michopoulos F, et al. Global metabolic profiling of animal and human tissues via UPLC-MS. *Nat Protoc* 2013;**8**:17-32.

12. Horai H, Arita M, Kanaya S, et al. MassBank: a public repository for sharing mass spectral data for life sciences. *J Mass Spectrom* 2010;**45**:703-714.

13. Sud M, Fahy E, Cotter D, et al. LMSD: LIPID MAPS structure database. *Nucleic Acids Res* 2007;**35**:D527-532.

14. Abdelrazig S, Safo L, Rance GA, et al. Metabolic characterisation of Magnetospirillum gryphiswaldense MSR-1 using LC-MS-based metabolite profiling. *RSC Adv* 2020;**10**:32548-32560.

15. Gagnebin Y, Tonoli D, Lescuyer P, et al. Metabolomic analysis of urine samples by UHPLC-QTOF-MS: Impact of normalization strategies. *Anal Chim Acta* 2017;**955**:27-35.

16. Thévenot EA, Roux A, Xu Y, Ezan E, Junot C. Analysis of the Human Adult Urinary Metabolome Variations with Age, Body Mass Index, and Gender by Implementing a Comprehensive Workflow for Univariate and OPLS Statistical Analyses. *J Proteome Res* 2015;**14**:3322-3335.

17. Xia J, Wishart DS. Web-based inference of biological patterns, functions and pathways from metabolomic data using MetaboAnalyst. *Nat Protoc* 2011;**6**:743-760.

18. Dunn WB, Broadhurst D, Begley P, et al. Procedures for large-scale metabolic profiling of serum and plasma using gas chromatography and liquid chromatography coupled to mass spectrometry. *Nat Protoc* 2011;**6**:1060-1083.

**Figures**

Figure S1 Study flow.


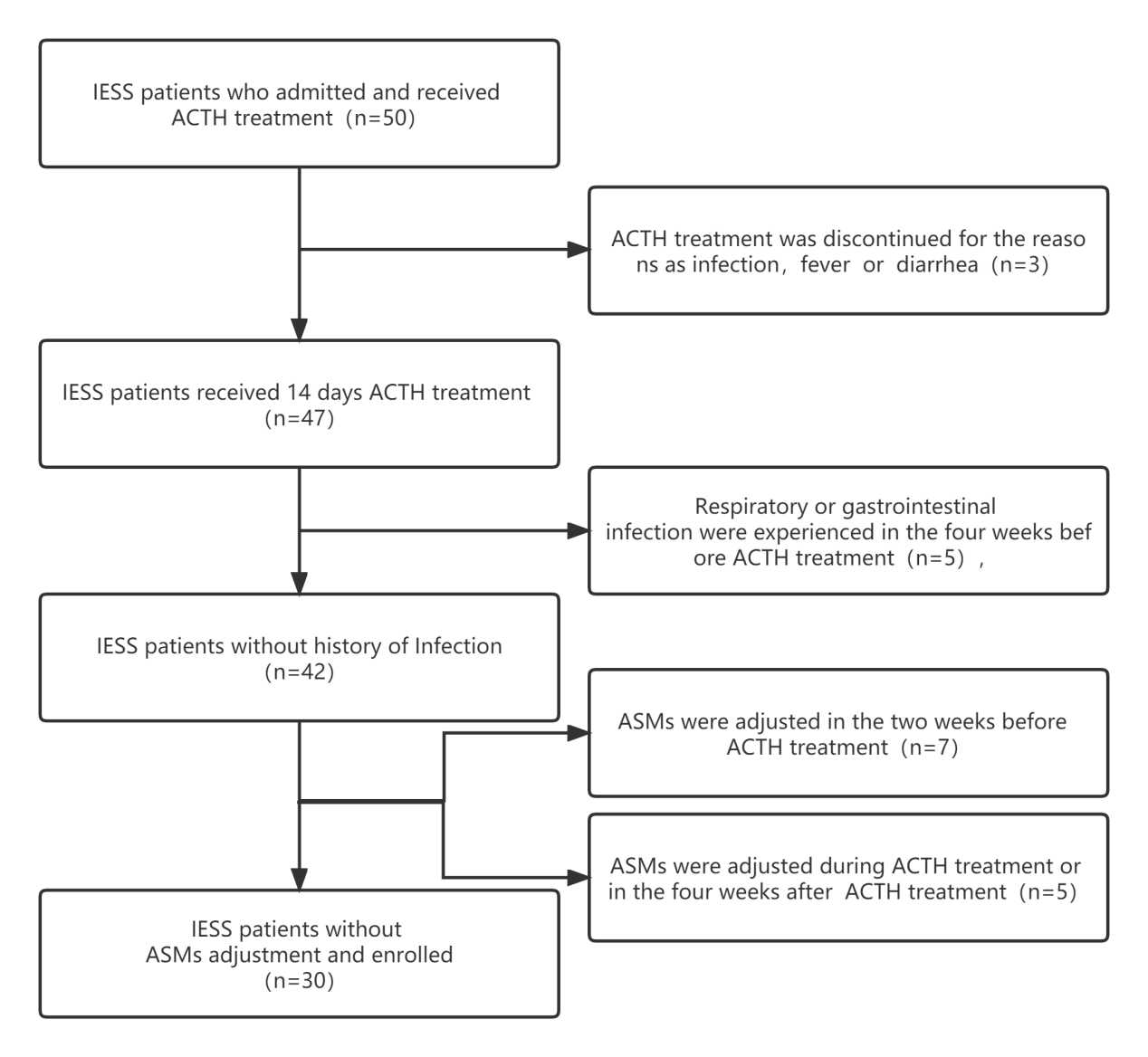


Figure S2 Analysis of α diversity between response and no response groups. a and b, comparison of species number differences; c-f, analysis by shannon, simpson, chao1 and ACE Indices between two groups, there were no significant differences in Shannon, Simpson, Chao1, and ACE indices between the two groups (ns, no significance; Red, no repsonse group, NRBA; blue, response group, RBA).


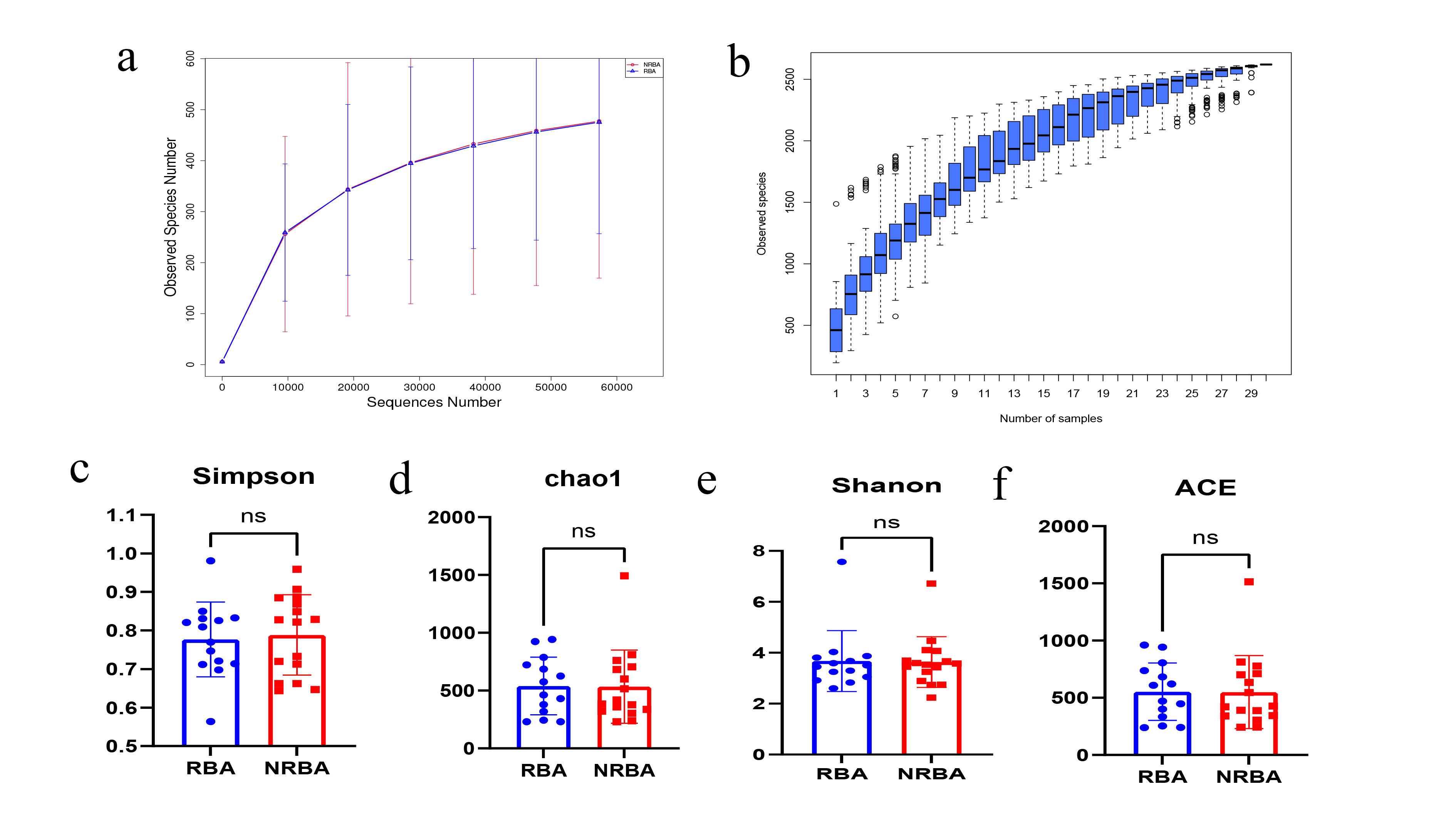


Figure S3 Analysis of β diversity between response and no response groups. There were no significant differences between the two groups in beta diversity(p<0.05). a. Principal Component Analysis; b. Principal Co-ordinates Analysis; c. Non-Metric Multi-Dimensional Scaling and d. Unweighted Pair-group Method with Arithmetic Means. (Red, no repsonse group, NRBA; blue, response group，RBA).


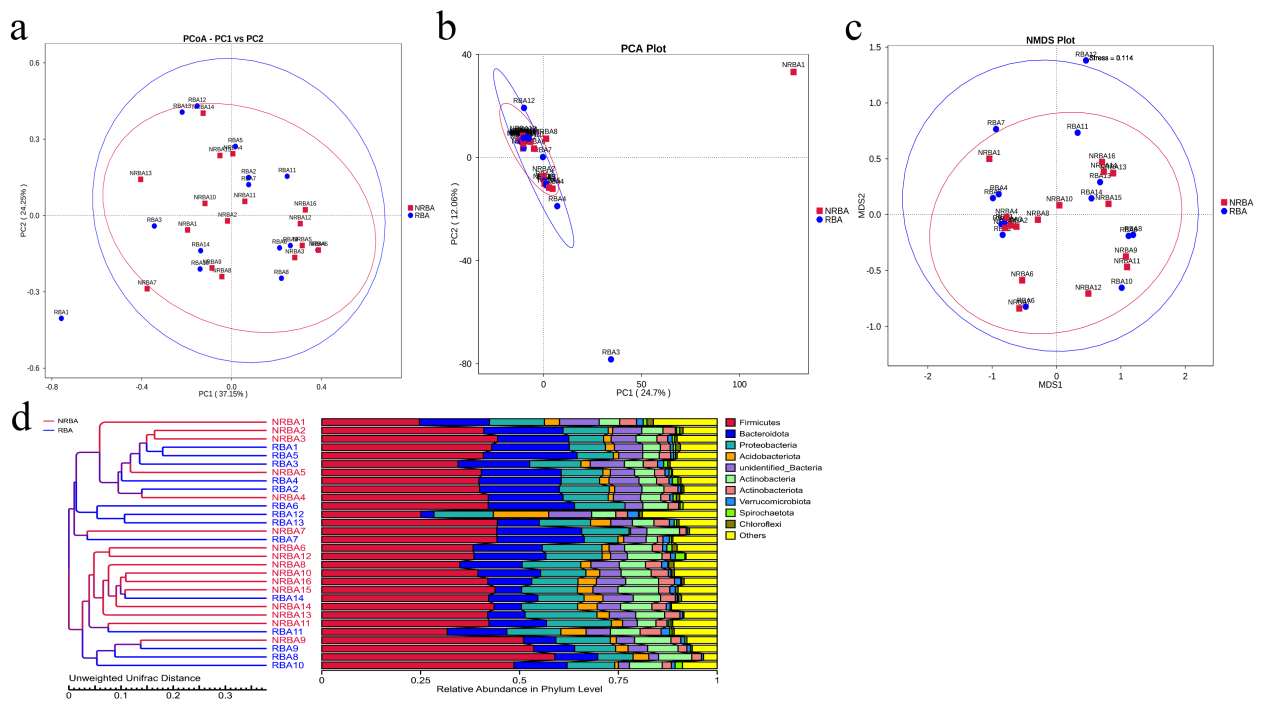


**Table**

| Table S1.The difference in bacterial genus and species between the response group and no response group(analysed by Metastat) | | | | | | | | |
| --- | --- | --- | --- | --- | --- | --- | --- | --- |
| Genus | mean(group1) | variance(group1) | standard error(group1) | mean(group2) | variance(group2) | standard error(group2) | p value | q value |
| Clostridioides | 0.001383957 | 5.48E-06 | 0.000585295 | 0.000236579 | 4.96E-08 | 5.95E-05 | 0.000999001 | 0.045200969 |
| Erysipelotrichaceae | 0 | 0 | 0 | 1.95E-05 | 5.33E-09 | 1.95E-05 | 0.000999001 | 0.045200969 |
| Acidisoma | 0 | 0 | 0 | 1.83E-05 | 4.68E-09 | 1.83E-05 | 0.000999001 | 0.045200969 |
| unidentified_Kapabacteriales | 1.39E-05 | 3.08E-09 | 1.39E-05 | 0 | 0 | 0 | 0.000332248 | 0.034830619 |
| Mycobacterium | 1.39E-05 | 1.25E-09 | 8.85E-06 | 0 | 0 | 0 | 0.000332248 | 0.034830619 |
| Thermomonas | 1.07E-06 | 1.82E-11 | 1.07E-06 | 1.59E-05 | 2.98E-09 | 1.46E-05 | 0.000545582 | 0.040023423 |
| Proteiniphilum | 1.49E-05 | 2.64E-09 | 1.28E-05 | 1.22E-06 | 2.08E-11 | 1.22E-06 | 0.001331578 | 0.046531264 |
| Chitinibacter; | 1.17E-05 | 2.20E-09 | 1.17E-05 | 0 | 0 | 0 | 0.001221648 | 0.045200969 |
| Iamia | 1.17E-05 | 2.20E-09 | 1.17E-05 | 0 | 0 | 0 | 0.001221648 | 0.045200969 |
| Bauldia | 1.39E-05 | 1.91E-09 | 1.09E-05 | 0 | 0 | 0 | 0.000332248 | 0.034830619 |
| Turneriella | 1.28E-05 | 1.57E-09 | 9.92E-06 | 0 | 0 | 0 | 0.000636302 | 0.040023423 |
| Chujaibacter | 0 | 0 | 0 | 1.34E-05 | 1.44E-09 | 1.02E-05 | 0.000228591 | 0.034830619 |
| Asteroleplasma | 0 | 0 | 0 | 1.22E-05 | 1.36E-09 | 9.87E-06 | 0.000489841 | 0.040023423 |
| Sharpea | 0 | 0 | 0 | 1.46E-05 | 1.43E-09 | 1.01E-05 | 0.000106675 | 0.034830619 |
| Gemmobacter | 1.17E-05 | 1.12E-09 | 8.35E-06 | 0 | 0 | 0 | 0.001221648 | 0.045200969 |
| KD3-10 | 1.17E-05 | 7.66E-10 | 6.92E-06 | 0 | 0 | 0 | 0.001221648 | 0.045200969 |
| Phocaeicola | 1.28E-05 | 9.13E-10 | 7.56E-06 | 0 | 0 | 0 | 0.000636302 | 0.040023423 |
| Phaselicystis | 1.49E-05 | 8.89E-10 | 7.45E-06 | 0 | 0 | 0 | 0.000173879 | 0.034830619 |
| Sepcies |  |  |  |  |  |  |  |  |
| Peptoclostridium_phage_p630P2 | 0.001380756 | 5.49E-06 | 0.000585671 | 0.000236579 | 4.96E-08 | 5.95E-05 | 0.000999001 | 0.032539626 |
| Odoribacter_splanchnicus | 0 | 0 | 0 | 0.000331698 | 1.54E-06 | 0.000331698 | 0.000999001 | 0.032539626 |
| Alistipes_inops | 0 | 0 | 0 | 9.39E-05 | 1.23E-07 | 9.39E-05 | 0.000999001 | 0.032539626 |
| Firmicutes_bacterium_M10-2 | 0 | 0 | 0 | 4.02E-05 | 1.98E-08 | 3.77E-05 | 0.000999001 | 0.032539626 |
| Lactobacillus_ruminis | 0 | 0 | 0 | 2.80E-05 | 9.09E-09 | 2.55E-05 | 0.000999001 | 0.032539626 |
| Faecalitalea_sp_Marseille-P3755 | 0 | 0 | 0 | 1.95E-05 | 5.33E-09 | 1.95E-05 | 0.000999001 | 0.032539626 |
| Pseudoalteromonas_phenolica | 1.60E-05 | 4.10E-09 | 1.60E-05 | 0 | 0 | 0 | 9.12E-05 | 0.032539626 |
| Methylobacterium_oxalidis | 1.49E-05 | 3.57E-09 | 1.49E-05 | 1.22E-06 | 2.08E-11 | 1.22E-06 | 0.001331578 | 0.038330433 |
| Bacteroidetes_bacterium_OLB10 | 1.39E-05 | 3.08E-09 | 1.39E-05 | 1.22E-06 | 2.08E-11 | 1.22E-06 | 0.002391042 | 0.043799537 |
| Coprococcus_eutactus | 2.13E-06 | 3.40E-11 | 1.46E-06 | 1.59E-05 | 3.52E-09 | 1.59E-05 | 0.002818401 | 0.049383281 |
| Sutterellaceae_bacterium_Marseille-P2968 | 0 | 0 | 0 | 1.34E-05 | 2.52E-09 | 1.34E-05 | 0.000228591 | 0.032539626 |
| Pinus_canariensis | 1.07E-05 | 1.82E-09 | 1.07E-05 | 0 | 0 | 0 | 0.002351832 | 0.043799537 |
| Kapabacteria_sp_59-99 | 1.07E-05 | 1.82E-09 | 1.07E-05 | 0 | 0 | 0 | 0.002351832 | 0.043799537 |
| bacterium_Ellin506 | 1.07E-05 | 1.82E-09 | 1.07E-05 | 0 | 0 | 0 | 0.002351832 | 0.043799537 |
| Lactobacillus_ingluviei | 0 | 0 | 0 | 1.10E-05 | 1.69E-09 | 1.10E-05 | 0.001049665 | 0.032539626 |
| Enterobacter_sp_Nj-68 | 0 | 0 | 0 | 9.76E-06 | 1.33E-09 | 9.76E-06 | 0.002249295 | 0.043799537 |
| Olsenella_scatoligenes | 0 | 0 | 0 | 9.76E-06 | 1.33E-09 | 9.76E-06 | 0.002249295 | 0.043799537 |
| Prevotella_sp_oral_clone_DA058 | 1.39E-05 | 1.29E-09 | 8.98E-06 | 0 | 0 | 0 | 0.000332248 | 0.032539626 |
| Candidatus_Saccharibacteria_bacterium_UB2523 | 0 | 0 | 0 | 1.22E-05 | 1.36E-09 | 9.87E-06 | 0.000489841 | 0.032539626 |
| Prevotella_sp_RS2 | 0 | 0 | 0 | 1.71E-05 | 1.93E-09 | 1.17E-05 | 0.000999001 | 0.032539626 |
| Aquaspirillum_sp_feline_oral_taxon_080 | 1.07E-05 | 1.20E-09 | 8.66E-06 | 0 | 0 | 0 | 0.002351832 | 0.043799537 |
| Phocaeicola_abscessus | 1.28E-05 | 9.13E-10 | 7.56E-06 | 0 | 0 | 0 | 0.000636302 | 0.032539626 |
| Pedobacter_panaciterrae | 1.39E-05 | 7.08E-10 | 6.65E-06 | 1.22E-06 | 2.08E-11 | 1.22E-06 | 0.002391042 | 0.043799537 |
