# Supplementary figures and images for: Abnormalities in Clostridioides and related metabolites before ACTH treatment may be associated with its efficacy in patients with infantile epileptic spasm syndrome
Source: CNS Neurosci Ther. 2023 Aug 8;30(1):e14398. doi: 10.1111/cns.14398 (PMC10805391; doi:10.1111/cns.14398)

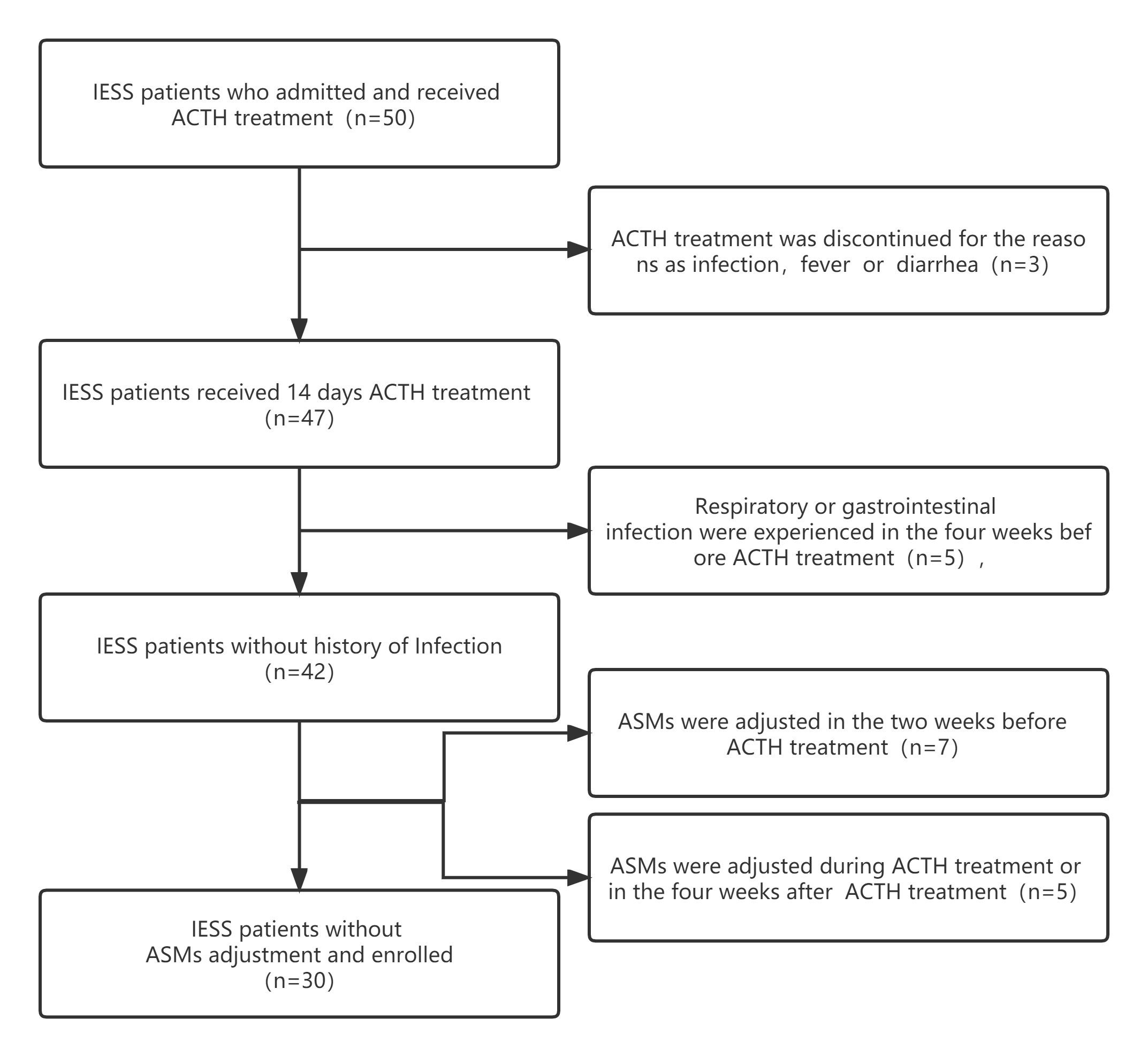

Supplement: Supplementary file 1 — Data S1. [file CNS-30-e14398-s001.zip › Figure S1_1.png]

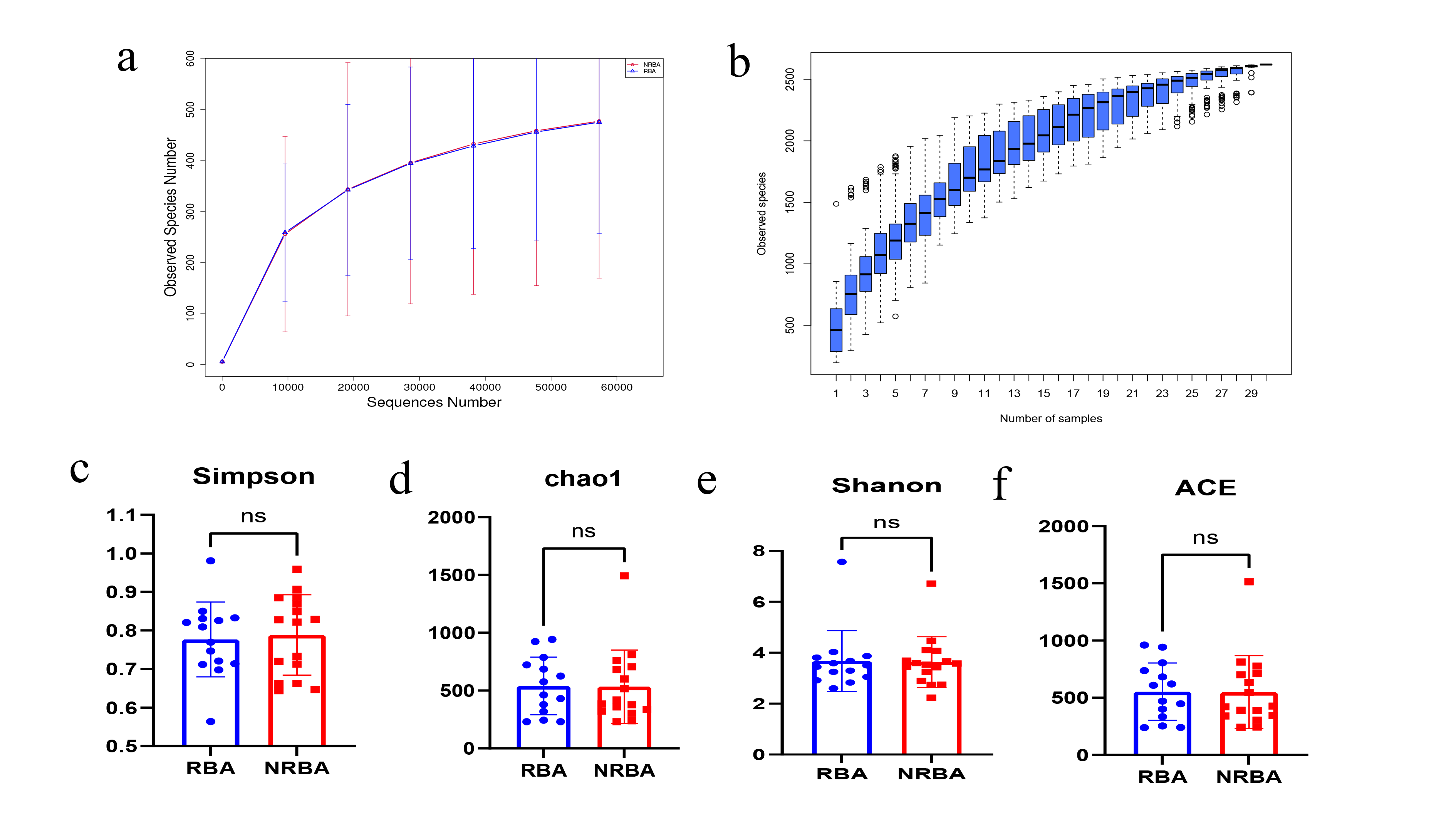

Supplement: Supplementary file 1 — Data S1. [file CNS-30-e14398-s001.zip › FigureS2 (2)_1.tif]

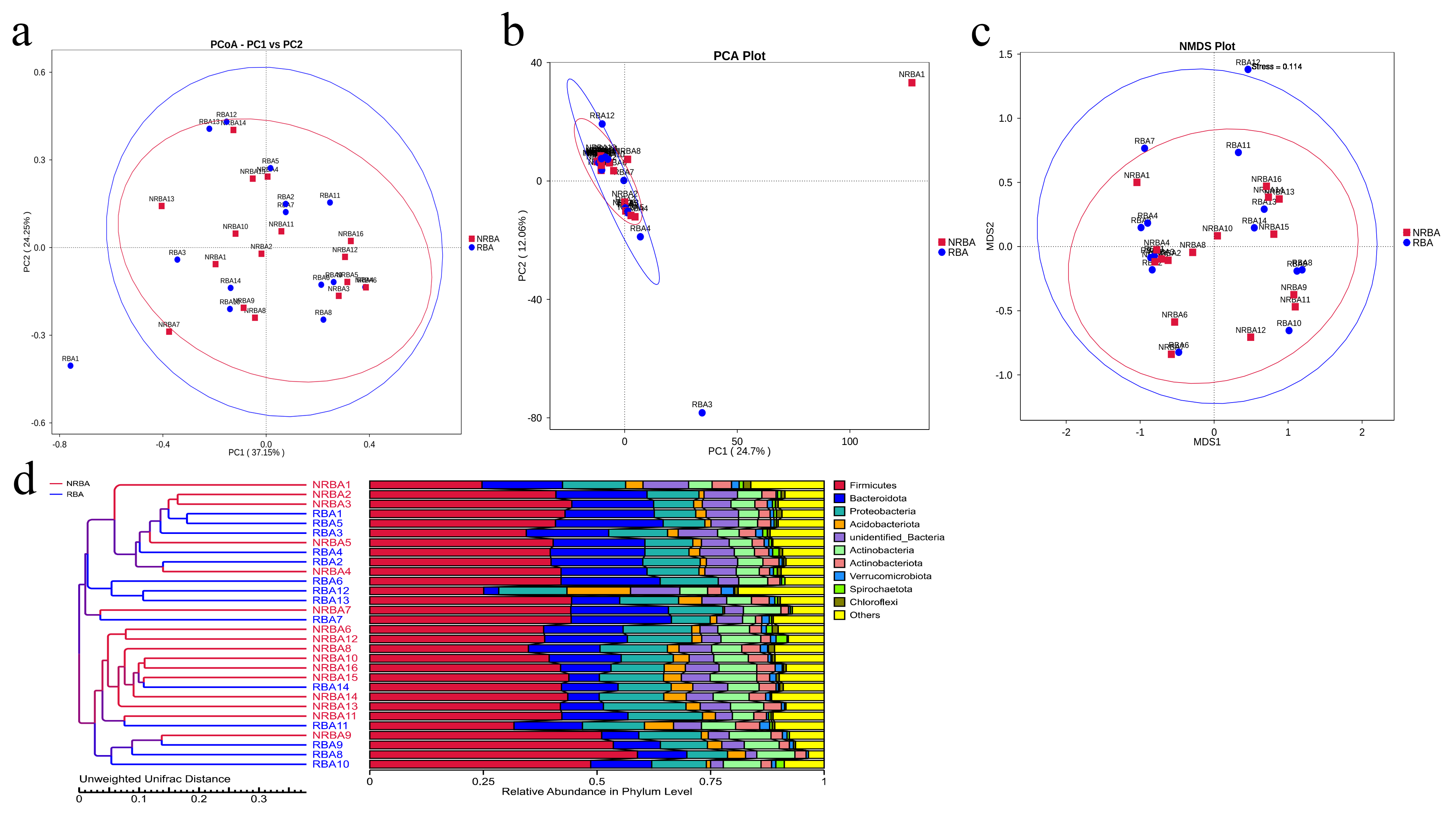

Supplement: Supplementary file 1 — Data S1. [file CNS-30-e14398-s001.zip › FigureS3_1.tif]
